# Parasite egg shedding rhythms are independent of feeding habits in a small shorebird host

**DOI:** 10.1017/S0031182026101851

**Published:** 2026-04

**Authors:** Simón Maldonado-Oyarzo, María Fernanda Sánchez-Méndez, Pablo Oyarzún-Ruiz, Luis Vargas-Chacoff, Juan G. Navedo, José O. Valdebenito

**Affiliations:** 1 Bird Ecology Lab, Instituto de Ciencias Marinas y Limnológicas, Universidad Austral de Chile, Valdivia, Chile; 2 Estación Experimental Quempillén, Facultad de Ciencias, Universidad Austral de Chile, Ancud, Chiloé, Chile; 3 Millennium Institute Biodiversity of Antarctic and Subantarctic Ecosystems (BASE), Universidad de Chile, Santiago, Chile; 4 Departamento de Microbiología, Facultad de Ciencias Biológicas, Universidad de Concepción, Concepción, Chile; 5 Laboratorio de Fisiología de Peces, Instituto de Ciencias Marinas y Limnológicas, Universidad Austral de Chile, Valdivia, Chile; 6 Centro FONDAP de Investigación en Dinámica de Ecosistemas Marinos de Altas Latitudes (IDEAL), Universidad Austral de Chile, Valdivia, Chile; 7 Departamento de Zoología, Facultad de Ciencias Naturales y Oceanográficas, Universidad de Concepción, Concepción, Chile

**Keywords:** circadian, egg shedding, endoparasite life cycle, feeding behaviour, tidal cycle, wader

## Abstract

Host–parasite coevolution resulted in parasites optimizing their life cycle to obtain the most benefit from the host’s behaviour. In passerines, for instance, some parasite groups have found means to match their egg shedding rhythms with the hours of the day when hosts are most active. In other bird groups, such as shorebirds, whose active times are not determined by day–night cycles but by other external factors such as tidal or lunar cycles, it is not yet known whether their endoparasites exhibit any predictable pattern in their egg shedding rhythms. Here we used a simple wild-caught (captive) system, to provide a first approximation of the parasite egg shedding patterns in wintering Rufous-chested dotterels (*Zonibyx modestus*). We collected faeces every 2 hours over 10 days until completing a 24-hour cycle, which was coupled with continuous video recordings to determine their feeding and drinking habits. Contrary to our expectation, we showed that parasite egg shedding followed a cyclic pattern, characterized by 2 peaks: one at midnight and another in the afternoon. Importantly, this shedding pattern was not related to the birds’ feeding or drinking habits. We discuss possible environmental and physiological cues that parasites might use to trigger egg production, including the potential influence of tidal cycles on our results.

## Graphical abstract

**Figure:**
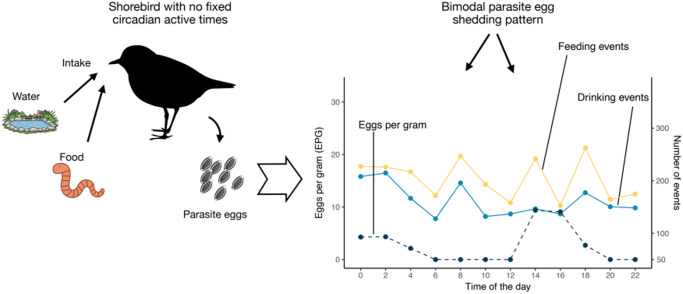

## Introduction

Parasites are widespread in nature and their importance in animal ecology and evolution is well recognized (Thomas et al., 2005). Some hosts and their parasites seem to have a long history of tight coevolution (Poinar and Boucot, 2006; Gandon et al., 2008), with parasites adapting to the systemic and behavioural characteristics of hosts in order to maximize their reproductive success (Poulin, 2007; Gomes et al., 2016). Infection with *Toxoplasma gondii* provides a good example of this adaptation since mice infected with this parasite lose their predator aversion behaviour, leading to a higher chance of predation by cats, the parasite’s definitive host and final stage in its life cycle (Boillat et al., 2020).

Another form of coevolution includes parasites adjusting the timing of their egg shedding, that is, the time of day when they maximize its release, to match the habits of the host in order to ensure their eggs are adequately released into the environment. This often occurs in parasites that release their eggs via the host’s gastrointestinal tract, i.e., endoparasites (Atkinson et al., 2008). We note that endoparasites include a wide range of species, including coccidian parasites that release oocysts instead of eggs, but in the interest of simplicity, we will use the term ‘eggs’ for both cases. Correct timing of egg shedding is crucial for successful completion of endoparasite life cycles, as they often have complex life cycles that may include one or more intermediate hosts. In parasite species exploiting hosts with a clear-cut circadian behaviour pattern, leading to day-night differences in their susceptibility to infection, egg shedding often shows circadian rhythms coupled with photoperiod (Kearn, 1986; Knight et al., 2018). This is typically found in Passeriformes and Galliformes, where egg shedding usually peaks during daytime matching the active diurnal phase of these hosts (López et al., 2007; Wongrak et al., 2015; Portugaliza et al., 2024). However, in parasite species affecting hosts with unpredictable circadian patterns, egg shedding could be triggered by alternative, specific cues. While there is little research available on this topic, it has been proposed that chemical compounds specific to the host mucus might have a role (Kearn, 1986), suggesting a possible indirect effect of food consumption as triggering cue for parasite egg release.

Shorebirds (Charadriiformes: sandpipers, plovers and allies) represent an excellent example of vertebrate hosts whose active times are often independent of circadian cycles. This is because active times of many species seem to be mainly governed by alternative factors like tidal or lunar cycles (Burger et al., 1977; Eberhart-Phillips, 2017; Fonseca et al., 2017). For instance, shorebirds frequently commute between different foraging and roosting habitats (McNeil et al., 1992). While a few studies suggest a high richness of gastrointestinal parasites in shorebirds, including coccidia, flukes, tapeworms, roundworms and spiny-headed worms (Siebert et al., 2012; Gutiérrez et al., 2017), it is still unclear whether parasite egg shedding in shorebirds follows any form of cyclic pattern. Moreover, it is also unknown which factors could explain patterns of parasite egg production in this bird group.

In this work we used a captive setup of wild-caught Rufous-chested dotterels (*Zonibyx modestus*; hereafter dotterels) to provide a preliminary assessment of whether the timing of their endoparasites’ egg shedding follows predictable rhythms and how these rhythms might relate to the birds’ foraging and roosting habits. This was carried out by determining the number of parasite eggs shed in bird droppings every 2 h in a 24 h cycle. This was complemented with continuous video recordings of the captive individuals, used to quantify their feeding and drinking frequencies over the same time cycle. Due to the lack of a fixed diurnal/nocturnal schedule in dotterels, we predicted that their parasite egg shedding rhythms lacked a distinctive pattern, contrary to what has been seen in diurnal birds (e.g. songbirds; López et al., 2007). Considering that it is possible that signals related to food or water consumption by the host could trigger parasite egg production (Kearn, 1986), we predicted that egg shedding rhythms might follow that of their feeding or drinking habits, because it could represent a signal that the host is on the correct environment for the parasite to release eggs and secure the continuity of its life cycle.

## Methods

### Animal capture and care

The Rufous-chested dotterel is a small shorebird (∼75 g) native to the Southern Cone of South America with a conservation status of Least Concern (IUCN, 2025). Dotterels were captured in August 2023, in their non-breeding grounds near Ancud, Chiloé Archipelago, Chile (41°53’11”S, 73°57’31”W). We chose these birds because they belonged to a well-monitored population that inhabits the area during the winter months. They routinely commute between their intertidal feeding areas at the shore and roosting areas located a few kilometres inland. Importantly, previous unpublished data from this population showed presence of gastrointestinal parasites, along with a previous record of *Porrocaecum falklandicus* for the species (Canaris et al., 2021). A total of 21 adult individuals (aged by plumage characteristics) were captured at night via spotlighting and hand-netting. Birds were ringed, morphologically measured, and then transported to nearby experimental facilities (30-mins drive) at Quempillén Experimental Field Station (Universidad Austral de Chile). Later a group of 4 birds was selected to take part in the experiment and thus housed in one aviary.

The indoor aviaries (5 × 2.5 × 2.5 m) have been especially built to host waterbirds, including shorebirds, and fitted with built-in, continuously running and independent saline and fresh-water systems. This allows birds to develop their natural maintenance behaviours (e.g. bathing, preening, etc.). The indoor temperature was fixed at 25ºC, in concordance with the thermoneutral zone of similar *Charadrius* species (Abad-Gómez et al., 2013). Birds had access to freshwater and food *ad libitum*. The photoperiod was kept natural, which for the latitude and time of the year corresponded to ∼10 h of light and ∼14 h of darkness (10 L:14D). Apart from the saline and freshwater pools, aviaries were also fitted with basic environmental enrichment elements, including clean rocks, plastic seaweed and a small container of sand (Figure S1).

Before the experiment, birds were acclimated for an 18-day period during which were fed with commercially available fishing worms to then progressively transition to dry pellets (Mazuri®). This ensures that each bird consumed food with the exact nutrition value as well as free of microorganisms that could confound the study. Importantly, by the time this experiment had commenced, all birds had been consuming only pellets for over 2 weeks. Fresh food and water were provided daily. Food was placed in 5 feeding trays, and water in one large tray, positioned opposite the feeding trays (Figure S1). Once a day, old trays were replaced with new ones containing approx. 50 g of pellets each. Importantly, when replacing food trays, these were generally half full, confirming constant food availability. After the experiment concluded, all birds were successfully released back into their natural environment.

### Study design

Plover species are known to be highly sensitive to handling and captivity. Captive conditions must therefore be optimal to ensure their well-being, which critically includes reducing disturbances to a minimum such as stepping into the aviaries. To comply with this minimized disturbance regime, we used a rotational sampling schedule over 10 days (29 August–07 September). We collected samples at different time points each day (e.g., 02:00 and 14:00 on Day 1; 04:00 and 16:00 on Day 2, etc.). This allowed us to accumulate data for all 12 two-hour intervals (00:00 to 22:00) by the end of the 10-day period, effectively constructing a complete composite 24-hour cycle. In parallel to the faecal sampling, we set-up video cameras with continuous recording during the last three 24-hour cycles of the experiment (see below).

### Faeces collection and egg shedding detection

Samples were collected using 3 black rubber mats (50 × 50 cm) placed directly on the aviary floor, underneath and around feeding trays, the areas where birds defecated the most and were seen roosting (Figure S1). Each mat was left in place for 2 hours before being replaced with a clean one. Faeces were stored in 1.5 ml microcentrifuge tubes with 70% ethanol. At each sampling moment, we collected between 1 and 3 full microcentrifuge tubes. Parasite egg detection was conducted using the Mini-FLOTAC® kit, which enables egg detection by filtering organic matter from the faeces to then allow flotation of eggs using a supersaturated solution. In our study we mixed faeces with ZnSO_4_ (solution specific gravity = 1.35) in a 1:10 ratio. We followed the standard recommendations by the manufacturer, with small modifications which allowed improve performance in bird droppings (see details in Cringoli et al., 2017; Lobos-Ovalle et al., 2021). Samples were observed at 40× magnification under a light microscope, and egg identification was conducted using taxonomic keys (Taylor et al., 2016; Mehlhorn, 2016). The concentration of eggs in faeces was standardized and expressed as number of eggs per gram of faeces (hereafter EPG; Cringoli et al., 2017).

### Estimating feeding behaviour

Animal behaviour was monitored using 2 small security cameras (‘Wyzecams’ or ‘Neos Smartcams’) with infrared light emitting diodes (LEDs), which were recording continuously for a period of three 24-hour cycles (∼72 h). These cameras were placed ∼50 cm above ground level and facing at different angles, aiming to obtain a complete view from inside the aviary. Footage was analysed in 24-hour or 48-hour ‘observations’ using the BORIS software (Friard and Gamba, 2016). The observations were conducted separately by bird, which were individually identified by a unique colour-ring combination. The analysis followed an ethogram that established a scoring system, where every time the target individual pecked from inside a food tray it corresponded to a feeding event. Likewise, every time the target individual dipped its bill in the water tray, it was noted as a drinking event. The data were exported in 24-hour portions and processed using a custom R code, as in Wanders et al. (2023). The resulting dataset had 17,504 feeding and drinking observations across the 4 birds in the aviary.

### Data analysis

To explore patterns of egg production throughout the day and their relationship with feeding and drinking habits, we employed a combination of generalized linear models (GLM) and generalized linear mixed models (GLMM) using R statistical software (v4.4.0; R Core Team, 2024). For model fitting, we utilized the glmmTMB R package, which supports the negative binomial family distribution and accommodates zero-inflated data (Brooks et al., 2017). Following the experimental design, we constructed 3 separate GLMs, each addressing a specific explanatory variable. The first model used EPG as the response variable and time (12 levels, i.e., every 2 hours from 00:00 to 22:00) as the explanatory variable. The other two models examined the number of feeding events and the number of drinking events as explanatory variables, respectively. To assess the general endoparasite shedding rhythm, the response variable (EPG) was calculated by pooling the counts of all detected parasite taxa. A negative binomial family distribution was used for all models, with a zero-inflation term included only in the analysis of EPG predicted by time. Additionally, we investigated whether feeding and drinking habits varied throughout the day. Here, three separate GLMMs were constructed: 2 models used feeding and drinking habits as response variables with time as the explanatory variable, while the third model examined the relationship between drinking and feeding habits to each other. All GLMMs were fitted with a negative binomial family distribution and included bird ID as a random-effect variable. Model suitability was assessed using the residual diagnostics tools from the DHARMa R package (Hartig, 2024).

## Results

**Figure 1.**
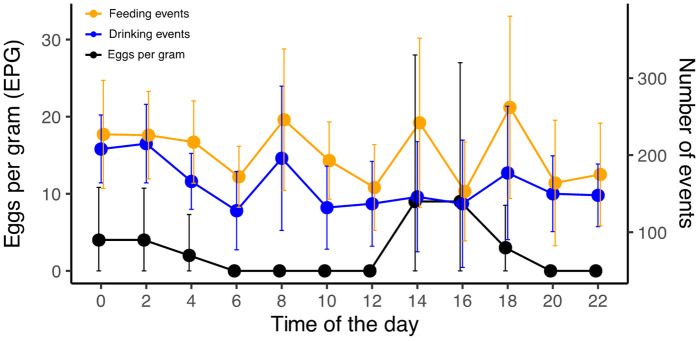
Food and water intake habits and parasite egg shedding rhythms throughout a 24-hour cycle in the Rufous-chested dotterel (*Zonibyx modestus*). The left *y*-axis shows the mean number of eggs per gram (in black) and the right *y*-axis shows the mean number of drinking (in blue) and feeding events (in Orange). Feeding and drinking events occurred at a consistent rate throughout the day whereas egg shedding had 2 peaks and completely stopped between 06:00–12:00 and 20:00–22:00. Most eggs shed corresponded to *Capillaria* parasites, except at 02:00, when Trematoda eggs were also released. Error bars indicate standard deviations. Note the different scales. Figure 1 long description.

Our analysis showed presence of eggs corresponding to two endoparasite morphotypes: nematodes (Phylum Nematoda, Family Capillariidae) and trematodes (Phylum Platyhelminthes, Class Trematoda). Capillarid eggs were the most frequently found across the study, whereas trematode eggs were detected in one sample during the nighttime peak (at 02:00). Helminth eggs (EPG) had a statistically significant variation throughout the 24-hour cycle (GLM; estimate = 0.048, standard error = 0.020, *z*-value = 2.363, *P*-value = 0.018), displaying 2 marked peaks that occurred approximately after midnight and then another one shortly after midday (Figure 1 and Table 1). We detected no egg release from faeces collected between 06:00 and 12:00, and then again from 20:00 to 22:00 (Figure 1). Feeding and drinking events were correlated to each other and while both events displayed several peaks at various times (Figure 1), only drinking events showed a statistically significant variation throughout the day (full results in Table S1). Notably, neither feeding nor drinking events significantly predicted EPG variation in the Rufous-chested dotterel (Table 1).

**Table 1.** Parasite eggs shed per gram of faeces (EPG) in relation to (a) time of the day, (b) feeding and (c) drinking events in the Rufous-chested dotterel (*Zonibyx modestus*) Table 1 long description.

	Estimate	Standard error	*z*-value	*P*-value
a) EPG in relation to time of the day* (*n* = 50)				
(Intercept)	−1.294	1.427	−0.907	0.364
Time **0**	3.881	1.444	2.688	0.007
Time **2**	3.881	1.444	2.688	0.007
Time **4**	**3.888**	**1.448**	**2.684**	**0.007**
Time 6	−1.684	4.514	−0.373	0.709
Time 8	−1.684	4.514	−0.373	0.709
Time 10	−1.684	4.514	−0.373	0.709
Time 12	−5.176	17.155	−0.302	0.763
Time **14**	4.979	1.436	3.468	0.001
Time **16**	4.921	1.436	3.427	0.001
Time **18**	**3.714**	**1.455**	**2.553**	**0.011**
Time 20	−13.60	941.9	−0.014	0.988
**b) EPG in relation to feeding events (*n* = 12)**				
(Intercept)	−0.290	2.453	−0.118	0.906
Feeding	0.006	0.012	0.507	0.612
**c) EPG in relation to drinking events (*n* = 12)**				
(Intercept)	0.407	2.739	0.148	0.882
Drinking	0.003	0.017	0.200	0.841

* Each sampling time was compared to time 22 (reference value), which had zero egg production.

## Discussion

This study provides the first preliminary insight into parasite egg shedding rhythms in an avian host whose active times are not determined by fixed circadian day-night cycles. We found that endoparasite egg shedding shows significant differences throughout the day, represented by a bimodal pattern, with one peak occurring after midnight and another one later in the early afternoon. But interestingly, this shedding rhythm was not associated with the birds’ feeding and drinking habits in captivity.

Shorebirds have been reported to carry a relatively high richness of gastrointestinal parasite groups (Gutiérrez et al., 2017) and *Porrocaecum falklandicus* is the only endoparasite previously reported in a Rufous-chested dotterel (though of unclear origin; see Canaris et al., 2021). In our study, the dotterels only showed presence of 2 parasites groups, identified as capillarids and trematodes. This relatively low diversity could in part be explained by the host’s immunocompetence, where variables like seasonal variations could up- or down-regulate immunity in birds (Hegemann et al., 2012; Sreedevi et al., 2016; Valdebenito et al., 2021). On the other hand, dietary and environmental changes associated with captivity could also drive variations in gastrointestinal parasite community in birds (Robinson et al., 2010; Aponte et al., 2014). But we note that while our study design was satisfactory to test our aims, a sample size of 4 individuals is arguably not sufficient for determining parasite richness. Nevertheless, our results represent the first record of helminth parasites in Rufous-chested dotterels from Chile.

The parasite egg-shedding strategy can vary substantially depending on the parasite species and whether the dotterels serve as the final host. Unfortunately, species-level taxonomic identification based on parasite egg morphology is limited. Capillarid parasites, the eggs from which dominated our findings, can have either a direct or an indirect life cycle, while trematodes usually have an indirect life cycle (Atkinson et al., 2008). Although speculative, this suggests that some of the eggs shed likely need to reach an intermediate host. Further studies are needed to uncover the actual implications of parasite life cycles on shorebird shedding rhythms.

Our results revealed a bimodal egg shedding peak within the 24-hour cycle in capillarid eggs and a nighttime release for trematodes. To our knowledge, there are no previous records of such egg shedding patterns in wild birds (Table 2). Most research on the topic has been conducted on Passeriformes and Galliformes, diurnal species in which egg shedding peaks during the daytime (Table 2 and references therein). One exception was presented by Taylor et al. (2018) on captive North Island brown kiwis (*Apteryx mantelli*), finding a shedding peak occurring at night and thus matching the nocturnal habits of these birds. Our results showed shedding peaks for both parasites occurring during the daytime and at night, which aligns with the flexible activity budgets of shorebirds, species whose foraging times are not strictly constrained by the light-dark cycle (Eberhart-Phillips, 2017). Altogether, this suggests that parasites have means to detect the moments when the host is active and release their eggs. This topic has been surprisingly understudied but it is possible that there are several mechanisms to achieve this, which could include variation in gastrointestinal activity, hormonal mediators or even environmental conditions, as has been shown in mammalian parasites (Valero et al., 2002; Ezenwa, 2004; Boelow et al., 2023). For instance, research on avian coccidia has proposed an important role for melatonin in mediating this process (Wild, 2004; Brandlmeier, 2006). However, a study on Snow Buntings (*Plectrophenax nivalis*) in the high Arctic challenged this view, as oocyst shedding patterns maintained the rhythmicity observed at lower latitudes even in the absence of darkness (Dolnik et al., 2011). Kearn (1986) suggested that in gastrointestinal protozoan parasites, chemical substances in the host’s mucus might play a role in the signalling process. However, considering that eating and drinking trigger mucus secretion throughout the gastrointestinal system, our results provide little support for the possible mucus-sensing mechanism proposed by Kearn (1986), at least in our dotterel–parasite captive system.

**Table 2.** Summary of shedding rhythms of (a) coccidian oocysts and (b) helminth eggs in birds. Most patterns were classified as early- or late-diurnal peaks (maximum shedding in morning or late afternoon/evening, respectively) or diurnal plateau (rapid morning increase sustained until evening). Less common patterns included a nighttime peak and the bimodal rhythm (one during the day and another at night) observed in the present study Table 2 long description.

Host species	Parasite group/species	Egg shedding rhythmicity	Enclosure	Reference
**a) Coccidia**				
*Plectrophenax nivalis*	*Isospora plectrophenaxia*	Late-diurnal peak	Free-living	Dolnik et al. (2011)
*Turdus merula*	*Isospora turdi*	Late-diurnal peak	Free-living	Filipiak et al. (2009)
*Saltator similis*	*Isospora similisi; I. saltatori; I. trincaferri; I. vanriperorum,*	Late-diurnal peak	Animal rescue centre	Coelho et al. (2013)
*Xanthomyza phrygia*	*Isospora lesouefi*	Late-diurnal peak	Zoo	Morin-Adeline et al. (2011)
*Apteryx mantelli*	*Eimeria* spp.	Nighttime peak	Outdoor aviaries	Taylor et al. (2018)
Temperate forest avian community	*Isospora* spp.	Late-diurnal peak	Free-living	Biard et al. (2022)
Tropical forest avian community	*Isospora* spp.	Diurnal plateau	Free-living (undergrowth)	Biard et al. (2022)
Tropical forest avian community	*Isospora* spp.	Late-diurnal peak	Free-living (open area)	Biard et al. (2022)
*Haemorhous mexicanus*	*Isospora* sp.	Late-diurnal peak	Free-living	Brawner and Hill (1999)
*Chloris chloris*	*Isospora* sp.	Late-diurnal peak	Free-living	Brown et al. (2001)
*Serinus serinus, Sylvia borin*	*Isospora*-like	Late-diurnal peak	Free-living	López et al. (2007)
*Turdus merula*	*Isospora* spp.	Late-diurnal peak	Free-living	Misof (2004)
*Sturnus unicolor*	*Isospora* sp.	Late-diurnal peak	Free-living	de la Peña et al. (2024)
**b) Helminths**				
*Alectoris rufa*	*Aonchotheca caudinflata*	Late-diurnal peak	Outdoor aviaries	Villanúa et al. (2006)
*Gallus gallus domesticus*	*Heterakis gallinarum*	Diurnal plateau	Poultry aviaries	Wongrak et al. (2015)
*Gallus gallus domesticus*	*Capillaria* spp.	Diurnal plateau	Poultry aviaries	Wongrak et al. (2015)
*Gallus gallus domesticus*	*Ascaridia galli*	Early-diurnal peak	Poultry aviaries	Wongrak et al. (2015)
*Gallus gallus domesticus*	*Heterakis gallinarum*	Late-diurnal peak	Poultry aviaries	Daş et al. (2019)
*Gallus gallus domesticus*	*Ascaridia galli*	Early-diurnal peak	Poultry aviaries	Portugaliza et al. (2024)
*Zonibyx modestus*	Capillariidae	Bimodal	Captive wild birds	This study
*CharadZonibyx modestus*	Trematoda	Nighttime peak	Captive wild birds	This study

The use of indoor aviaries allows us to control for numerous factors that would otherwise be sources of unwanted variation in this kind of studies. However, among the limitations of aviaries for shorebirds, we highlight the evident absence of tides. The feeding habits of shorebirds in their natural habitat are often closely linked to daily tidal cycles, as tides determine availability of intertidal feeding areas (Fonseca et al., 2017). During high tide, the foraging area decreases or even becomes unavailable, to then reappear when the tide is low (Turpie and Hockey, 1993; Fonseca et al., 2017; Navedo et al., 2019). Therefore, during high tide, shorebirds, particularly during wintering, tend to move to roosting areas where they don’t usually feed (Turpie and Hockey, 1993; Navedo and Herrera, 2012). The timing of high and low tides is constantly changing due to the approx. 25-hour periodicity of the moon’s daily cycle, resulting in each low tide being followed by a high tide after 6 hours and some minutes. This causes the timing of tides to shift progressively each day, making it possible for low and high tides to occur at any time of the day over the course of several days (Calle et al., 2016).

It is therefore interesting that our results showed a strong bimodality in egg shedding, separated by approximately 12 hours, which matches the occurrence of low tide in the natural environment. In other words, although it was not the aim of our study, it is possible that the egg shedding rhythms in our dotterels occur in such bimodality because parasites adapt to match the usual feeding windows of their host. A possible counterargument could note that our sample collection was spread over 10 days, which should result in a somewhat ample range of egg shedding peaks. However, each low and high tide peak advances by just a couple of minutes within a 24-hour cycle, making our results perfectly plausible for the given sampling timeframe. Future studies should investigate whether parasite egg shedding times match the actual moments when the intertidal feeding areas become available (Fonseca et al., 2017). Although, we remark on the fact that the foraging pattern following tides is not universal across shorebird species and may not be found throughout their entire annual cycle. For example, tides may have less influence on the daily foraging activity in species that obtain food in supratidal areas and would not affect migratory birds during their breeding period in inland areas (Holmes, 1966; Larsen, 1993). As is the case with our study model, which migrates to southern Patagonia to breed in continental areas from September to March (Kusch and Marín, 2004; Faria et al., 2023). Thus, it is possible that egg shedding patterns may vary between seasons in shorebirds (but see Dolnik et al., 2011).

In conclusion, though with limited sample size, we found significant differences in egg shedding throughout the day, with peaks occurring twice a day in a bimodal pattern: one occurring during the night and the other in the afternoon. We did not find a relationship between feeding or drinking habits and egg shedding, but while an experimental/captive setup is advantageous in some aspects, it may have limitations in replicating the birds’ natural environment, which could obscure some signals. Nevertheless, the literature has so far coincided with our results in that it is still unknown which environmental or physiological cues parasites use to synchronize and trigger egg shedding. A bimodal parasite egg shedding pattern in shorebirds that mostly match their active times with the also bimodal tidal cycles, is intriguing. Future studies should further investigate this relationship and explore whether this shedding pattern persists during the breeding season, when dotterels nest inland and spend half their time sitting on the nest. Understanding which parasite species are infecting the host is also crucial to fully comprehend the potential egg production strategies. We emphasize the need for further research to elucidate the precise mechanisms influencing egg-shedding patterns in hosts that do not adhere to fixed light/darkness schedules, such as shorebirds.

## Supporting information

10.1017/S0031182026101851.sm001 Maldonado-Oyarzo et al. supplementary material Maldonado-Oyarzo et al. supplementary material

## Data Availability

The full dataset and R code are available on FigShare.com (10.6084/m9.figshare.26125669).

## Supplementary material

The supplementary material for this article can be found at https://doi.org/10.1017/S0031182026101851

## Figure 1 Long description

Eggs per gram (EPG) A single line graph with three series and error bars. The x-axis is labeled Time of the day, ranging from 0 to 22 in increments of 2. The left y-axis is labeled Eggs per gram (EPG), ranging from 0 to 6 in increments of 2. The right y-axis is labeled Number of events, ranging from 0 to 300 in increments of 100. A legend at the upper left lists: Feeding events, Drinking events, Eggs per gram. Eggs per gram series: Values are about 2 at 0, about 2 at 2, about 1 at 4, 0 at 6, 0 at 8, 0 at 10, 0 at 12, about 2 at 14, about 2 at 16, about 2 at 18, 0 at 20 and 0 at 22. Vertical error bars are shown at each time point. Feeding events series: Values are about 200 at 0, about 200 at 2, about 200 at 4, about 150 at 6, about 200 at 8, about 150 at 10, about 200 at 12, about 150 at 14, about 250 at 16, about 150 at 18, about 250 at 20 and about 150 at 22. Vertical error bars are shown at each time point. Drinking events series: Values are about 200 at 0, about 200 at 2, about 150 at 4, about 100 at 6, about 150 at 8, about 100 at 10, about 100 at 12, about 100 at 14, about 200 at 16, about 150 at 18, about 150 at 20 and about 150 at 22. Vertical error bars are shown at each time point.

Navigate back to Figure 1.

## Table 1 Long description

The table reports model results for parasite eggs per gram of faeces, tested against time of day, feeding events, and drinking events. For time of day with 50 samples, several times show statistically significant higher egg counts compared with the reference time: times 0, 2, and 4 have similar positive estimates with p values around 0.007; times 14 and 16 are also strongly positive with p values around 0.001; and time 18 is positive with a p value around 0.011. Times 6, 8, 10, and 12 are not statistically different from the reference, with negative estimates and large uncertainty. Time 20 has an extremely large standard error and a non-significant p value, indicating an unstable estimate. For feeding events with 12 samples, the feeding effect estimate is near zero and not statistically significant, and the intercept is also not significant. For drinking events with 12 samples, the drinking effect estimate is near zero and not statistically significant, and the intercept is not significant. Overall, time of day shows clear periods of higher egg shedding, while feeding and drinking do not show detectable associations in these small event-based samples.

Navigate back to Table 1.

## Table 2 Long description

The table compiles published observations of daily rhythms in parasite propagule shedding by birds, split into coccidian oocysts and helminth eggs, with host species, parasite taxon, housing context, and citation. For coccidia, late-diurnal peaks dominate across many hosts and settings, including multiple Isospora records in free-living birds and in zoo or rescue contexts. Exceptions among coccidia include a nighttime peak for Eimeria in Apteryx mantelli and a diurnal plateau reported for a tropical forest bird community in undergrowth habitat, while the same community in open areas shows a late-diurnal peak. For helminths, poultry studies report diurnal plateaus for Heterakis gallinarum and Capillaria species, and early-diurnal peaks for Ascaridia galli, alongside a late-diurnal peak for Heterakis gallinarum in another study. The present study adds less common helminth patterns in captive wild birds: a bimodal rhythm for Capillariidae and a nighttime peak for trematodes. Comparisons should be interpreted cautiously because hosts, parasite identification level, and enclosure conditions vary across studies.

Navigate back to Table 2.
