# Supplementary material for: Parasite egg shedding rhythms are independent of feeding habits in a small shorebird host
Source: Parasitology. 2026 Mar 25;153(5):667–73. doi: 10.1017/S0031182026101851 (PMC13315208; doi:10.1017/S0031182026101851)
Supplement: Maldonado-Oyarzo et al. supplementary material [file S0031182026101851sup001.pdf]

Table S1 Model results of the relationship between feeding and drinking habits to each other, and how (b) feeding and (c) drinking habits might be predicted by time of the day in captive Rufous-chested dotterel (*Charadrius modestus*)

	**Estimate**	**Stand. error**	**Z-value**	**P-value**
**a) Feeding habits (n = 48)**				
intercept	4.891	0.169	28.936	<0.001
drinking habits	0.002	0.001	3.083	0.002
Random term				
	variance	stand. deviation		
bird ID	0.050	0.223		
**b) Feeding habits (n = 48)**				
intercept	5.381	0.166	32.343	<0.001
time	-0.010	0.005	-1.872	0.061
Random term				
	variance	stand. deviation		
bird ID	0.092	0.303		
**c) Drinking habits (n = 48)**				
intercept	5.203	0.149	34.983	<0.001
time	-0.014	0.006	-2.358	0.018
Random term				
	variance	stand. deviation		
bird ID	0.065	0.255		

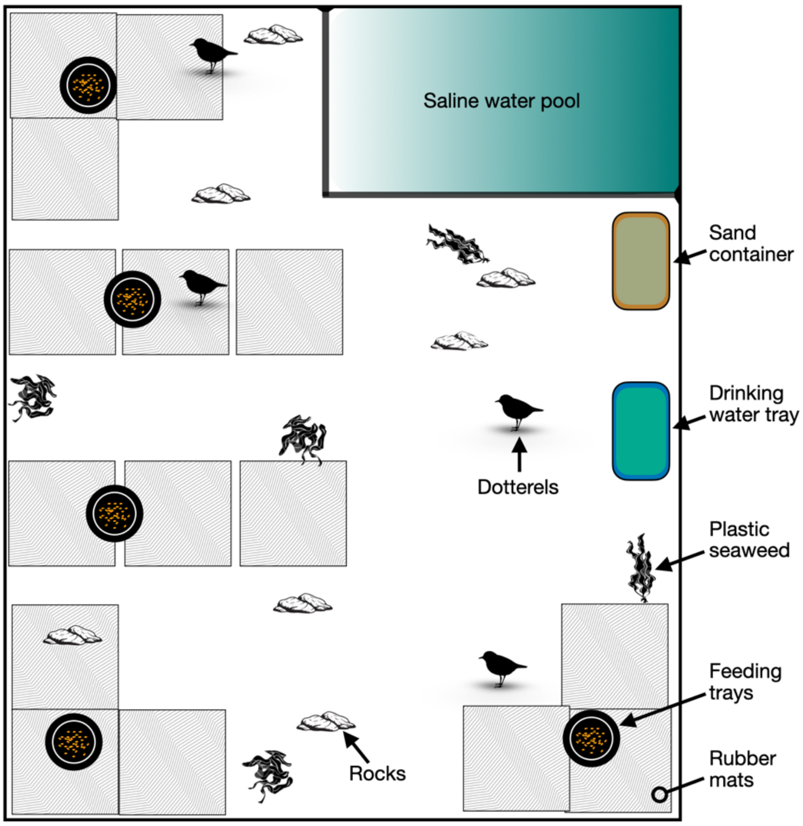

Figure S1 Schematic diagram of the experimental aviary layout (5 × 2.5 m). The enclosure housed four Rufous-chested dotterels (*Charadrius modestus*) and included environmental enrichment (sand, rocks and plastic seaweed). Five feeding trays were provided for the four birds (one surplus tray) to minimize competition, alongside a drinking water tray
